# Sequence-specific targeting of *Caenorhabditis elegans* C-Ala to the D-loop of tRNA^Ala^

**DOI:** 10.1016/j.jbc.2023.105149

**Published:** 2023-08-09

**Authors:** Titi Rindi Antika, Kun Rohmatan Nazilah, Dea Jolie Chrestella, Tzu-Ling Wang, Yi-Kuan Tseng, Sun-Chong Wang, Hsin-Ling Hsu, Shao-Win Wang, Tsung-Hsien Chuang, Hung-Chuan Pan, Jia-Cherng Horng, Chien-Chia Wang

**Affiliations:** 1 Department of Life Sciences, National Central University, Taoyuan, Taiwan; 2 Graduate Institute of Mathematics and Science Education, National Tsing Hua University, Hsinchu City, Taiwan; 3 Graduate Institute of Statistics, National Central University, Taoyuan, Taiwan; 4 Department of Biomedical Sciences and Engineering, National Central University, Taoyuan, Taiwan; 5 Institute of Molecular and Genomic Medicine, National Health Research Institutes, Miaoli, Taiwan; 6 Immunology Research Center, National Health Research Institutes, Miaoli, Taiwan; 7 Department of Neurosurgery, Taichung Veterans General Hospital, Taichung, Taiwan; 8 Department of Chemistry, National Tsing Hua University, Hsinchu, Taiwan

**Keywords:** aminoacyl-tRNA synthetase, *Caenorhabditis elegans*, coevolution, DNA-binding domain, translation, tRNA-binding domain

## Abstract

Alanyl-tRNA synthetase retains a conserved prototype structure throughout its biology. Nevertheless, its C-terminal domain (C-Ala) is highly diverged and has been shown to play a role in either tRNA or DNA binding. Interestingly, we discovered that *Caenorhabditis elegans* cytoplasmic C-Ala (Ce-C-Ala_c_) robustly binds both ligands. How Ce-C-Ala_c_ targets its cognate tRNA and whether a similar feature is conserved in its mitochondrial counterpart remain elusive. We show that the N- and C-terminal subdomains of Ce-C-Ala_c_ are responsible for DNA and tRNA binding, respectively. Ce-C-Ala_c_ specifically recognized the conserved invariant base G^18^ in the D-loop of tRNA^Ala^ through a highly conserved lysine residue, K934. Despite bearing little resemblance to other C-Ala domains, *C. elegans* mitochondrial C-Ala robustly bound both tRNA^Ala^ and DNA and maintained targeting specificity for the D-loop of its cognate tRNA. This study uncovers the underlying mechanism of how *C. elegans* C-Ala specifically targets the D-loop of tRNA^Ala^.

An aminoacyl-tRNA synthetase (aaRS) attaches a specific amino acid or its precursor to one of its cognate tRNAs to form an aminoacyl-tRNA. This charged tRNA is then delivered to the ribosome for polypeptide synthesis (1). Therefore, aminoacyl-tRNA plays a key role in DNA translation—the expression of genes to make proteins. Eukaryotes normally possess two distinct sets of aaRSs, one functioning in the cytoplasm and the other in mitochondria (2, 3, 4). Many eukaryotic aaRSs acquire noncatalytic N- or C-terminal appended domains to expand their nontranslational functions during evolution. However, AlaRS retains a conserved prototype structure across all three domains of life (5). This prototype structure consists of four domains: the catalytic domain, the tRNA-recognition domain, the editing domain, and the C-terminal alanyl-tRNA synthetase (C-Ala) domain (6) (Fig. 1*A*). Unlike the other three domains, the C-Ala domain is highly diverse in sequences across species. In prokaryotes, C-Ala is important not only for aminoacylation and editing but also for dimerization (7). However, in humans, this domain is dispensable for aminoacylation and binds DNA instead (8). Interestingly, the nematode C-Ala plays a significant role in aminoacylation and tRNA binding, but not in dimerization (9). C-Ala consists of an amino-terminal helical and a carboxyl-terminal globular subdomain. The helical subdomain mediates dimerization by forming a helix–loop–helix zipper, whereas the globular subdomain presents a positively charged surface suitable for tRNA binding (8, 10).

**Figure 1:**
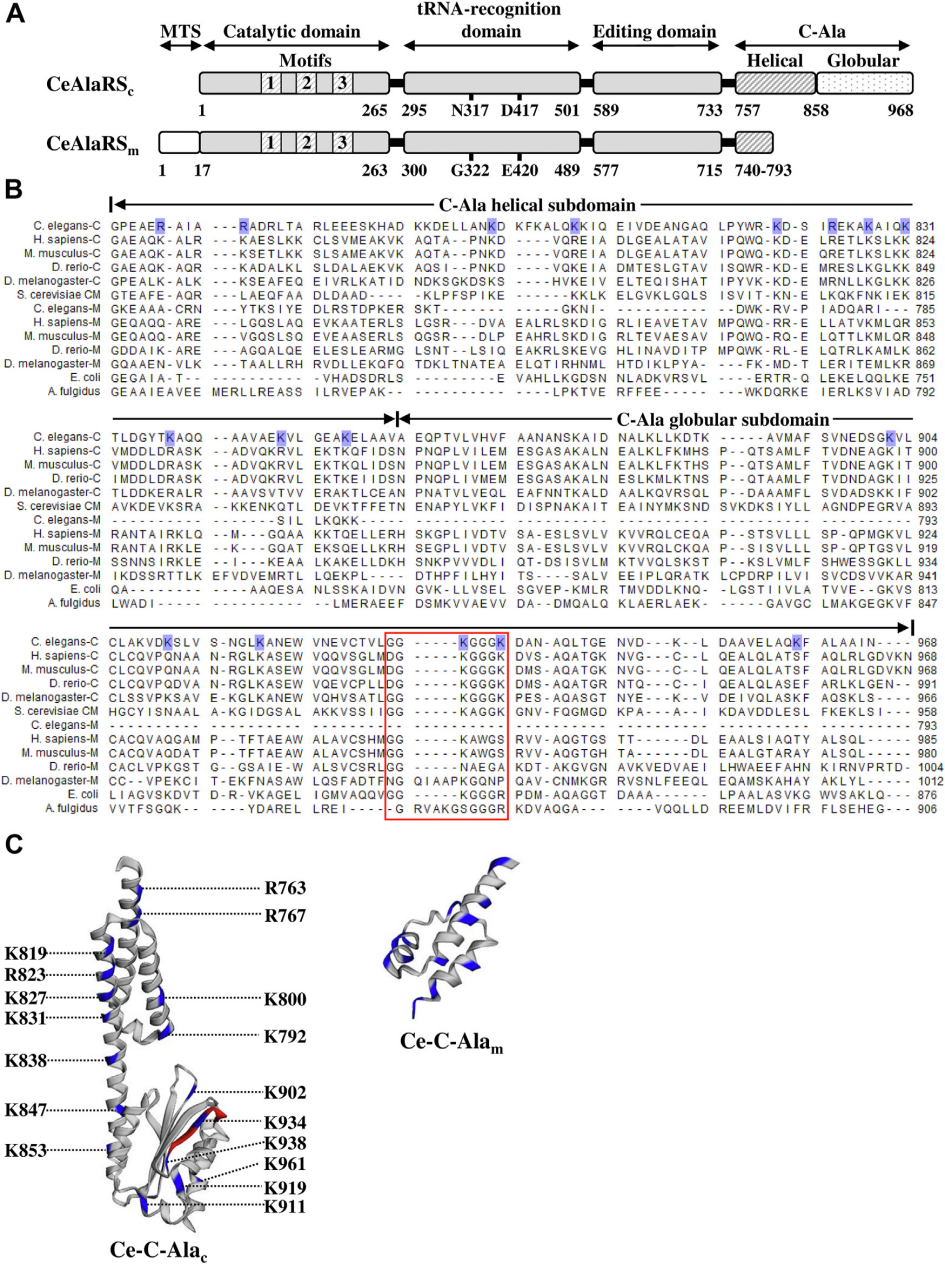
**C-Ala is highly diverged in sequence.***A*, modular organization of CeAlaRS_c_ and CeAlaRS_m_. The relative positions of the functional domains and motifs in the CeAlaRS isoforms are labeled. *B*, sequence alignment of C-Ala domains. C-Ala consists of an N-terminal helical and a C-terminal globular subdomain. Positively charged amino acid residues that are predicted to be involved in DNA or tRNA binding by Ce-C-Ala_c_ are highlighted in *blue*. A conserved GG-rich segment in the globular subdomain is boxed in *red*. *C*, a predicted tertiary structure of Ce-C-Ala_c_ and Ce-C-Ala_m_. The positively charged amino acid residues that are highlighted in (*B*) are colored in *blue*, and the GG-motif is colored in *red*. C-Ala, C-terminal domain of alanyl-tRNA synthetase; CeAlaRS_c_, cytoplasmic alanyl-tRNA synthetase of *C. elegans*; CeAlaRS_m_, mitochondrial alanyl-tRNA synthetase of *C. elegans*; Ce-C-Ala_c_, *C. elegans* cytoplasmic C-Ala; Ce-C-Ala_m_, *C. elegans* mitochondrial C-Ala.

A prototypic C-Ala domain consists of an N-terminal helical subdomain and a C-terminal globular subdomain. However, *Caenorhabditis elegans* mitochondrial C-Ala differs from the norm by having only one forth the size of a regular C-Ala domain. Moreover, C-Ala domains from prokaryotes and humans appear to play distinct roles. *Escherichia coli* C-Ala strongly prefers tRNA^Ala^ (7), whereas human cytoplasmic C-Ala strongly prefers DNA (8, 10). Strikingly, *C. elegans* cytoplasmic C-Ala (Ce-C-Ala_c_) robustly binds both ligands (9). In addition, fusion of Ce-C-Ala_c_ to the *C. elegans* mitochondrial AlaRS (containing a short C-Ala domain with only ∼50 amino acid residues) enables the enzyme to target and charge the elbow-containing tRNA^Ala^. Moreover, Ce-C-Ala_c_ binds various tRNAs with appreciable affinity, albeit with a distinct preference for tRNA^Ala^ (9). It remains unclear how Ce-C-Ala_c_ specifically targets the tRNA elbow. We showed herein that Ce-C-Ala_c_ specifically recognizes the conserved invariant base G^18^ in the D-loop of tRNA^Ala^ through a highly conserved lysine residue, K934. Despite C. elegans mitochondrial C-Ala (Ce-C-Ala_m_) bearing little resemblance to Ce-C-Ala_c_, it maintained targeting specificity for the D-loop of its cognate tRNA (as AAUAA). This study highlights not only the functional conservation but also the underlying mechanism of tRNA^Ala^ recognition by C-Ala.

## Results

### *C. elegans* cytoplasmic and mitochondrial C-Ala domains share little similarity

Except for *C. elegans* mitochondrial AlaRS, which possesses a C-Ala of only 54 amino acids, AlaRSs from *E. coli* to humans possess C-Ala domains of approximately 200 amino acids (Fig. 1*A*). In contrast to the enzyme’s other three domains, C-Ala is highly diverged among species (Figs. 1*B* and S1). For example, C-Ala from *C. elegans* cytoplasm (a higher eukaryote) shares only 21 to 42% similarity (identity plus conservative substitutions) with those from *S. cerevisiae* (a low eukaryote), *E. coli* (a bacterium), and *Archaeoglobus fulgidus* (an archaeon), whereas human mitochondrial C-Ala shares even less similarity with those C-Ala domains (Fig. S1). Paradoxically, paralogous C-Ala domains also share low similarity. Most strikingly, although Ce-C-Ala_m_ shares little sequence similarity with Ce-C-Ala_c,_ it forms a predicted structure somewhat resembling the N-terminal part (consisting of the first two helices) of the helical subdomain of Ce-C-Ala_c_. The protein models were obtained from AlphaFold Protein Structure Database (11, 12), and the amino acid residues that might be involved in tRNA or DNA binding were highlighted in different colors using EzMol 2.1 (http://www.sbg.bio.ic.ac.uk/ezmol/) (13) (Fig. 1*C*). Despite the extensive sequence divergence, C-Ala domains from eukaryotes were still clustered into a monophyletic group in phylogenetic analysis (Fig. S2) (14, 15). However, unlike the full-length AlaRS (9), the eukaryotic C-Ala group showed almost equally low affinity for the bacterial and archaeal C-Ala groups (compare Figs. S2 and S3).

In contrast to the N subdomain of *E. coli* C-Ala, which consists of two long α-helices with a linker between them, forming a helix–loop–helix zipper, the N subdomain of Ce-C-Ala_c_ contains an extra α-helix between the two α-helices (Fig. 1*C*). In addition, many positively charged amino acid residues (colored in *blue*) found in this subdomain are conserved in human cytoplasmic C-Ala, but not as many (or even none) are conserved in its counterparts from yeast, mitochondria, and prokaryotes (Fig. 1*B*). These positively charged amino acid residues are thought to take part in DNA binding (8, 9). Like the C subdomain of *E. coli* C-Ala, the C subdomain of Ce-C-Ala_c_ folds into a globular structure and tightly packs against the proximal helix of the N subdomain, with one positively charged surface facing outward (by the contributions of K902, K911, K919, K934, K938, and K961) (colored in *blue*) (Fig. 1*C*). Notably, all these positively charged amino acid residues are conserved in the otherwise diverged *E. coli* C-Ala but not in other C-Ala domains examined (Fig. 1*B*). This positively charged surface was shown to take part in tRNA binding (8, 10). Aligned behind this charged surface is a GG-rich segment (^932^GGKGGGK^938^) (colored in *red*), which forms a β-strand and is highly conserved from *E. coli* to humans (Fig. 1, *B* and *C*).

### The helical and globular subdomains of Ce-C-Ala_c_ prefer DNA and tRNA^Ala^, respectively

**Figure 2:**
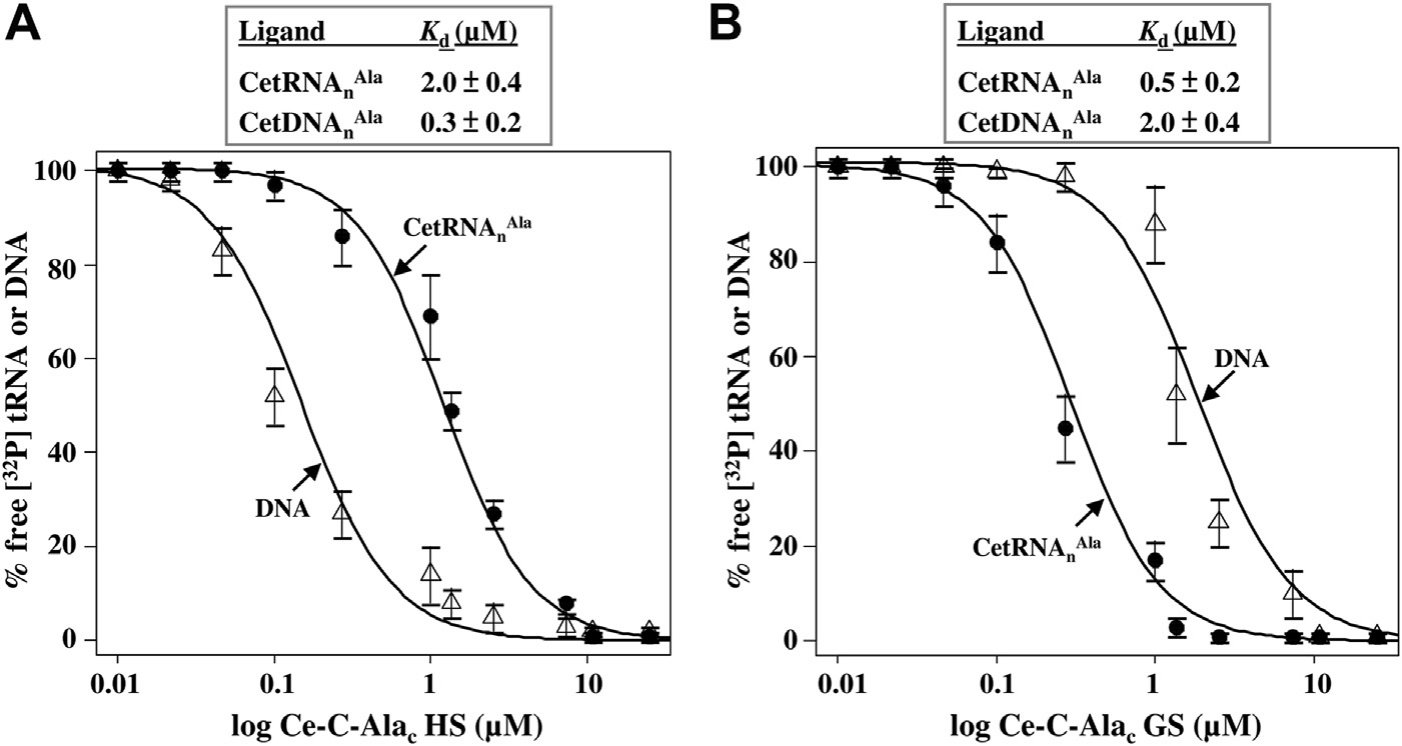
**The tRNA- and DNA-binding affinities of Ce-C-Ala**_**c**_**.** The tRNA- and DNA-binding affinities of the (*A*) helical (HS) and (*B*) globular (GS) subdomains of Ce-C-Ala_c_ were determined by an EMSA with protein concentrations ranging from 32 to 0.125 μM. The ^32^P-labeled CetRNA_n_^Ala^ and CetDNA_n_^Ala^ are shown in each panel. The equilibrium response at each protein concentration was fitted to a single-site binding model. Ce-C-Ala_c_, *Caenorhabditis elegans* cytoplasmic C-Ala; CetDNA_n_^Ala^, *C. elegans* cytoplasmic tDNA^Ala^; CetRNA_n_^Ala^, *C. elegans* cytoplasmic tRNA^Ala^.

To map the DNA- and tRNA-binding sites in Ce-C-Ala_c_, we tested the helical and globular subdomains individually using EMSA. We used *C. elegans* cytoplasmic tRNA^Ala^ (CetRNA_n_^Ala^) and CetDNA_n_^Ala^ as the ligands for this assay. CetDNA_n_^Ala^ is a double-stranded DNA fragment encoding CetRNA_n_^Ala^. As shown in Figure 2, the helical subdomain preferred DNA over tRNA^Ala^ (with *K*_*d*_ values of 0.3 μM for DNA and 2.0 μM for tRNA^Ala^). In contrast, the globular subdomain preferred tRNA^Ala^ over DNA (with *K*_*d*_ values of 2.0 μM for DNA and 0.5 μM for tRNA^Ala^). This result suggests that the helical and globular subdomains are mainly responsible for DNA and tRNA binding, respectively.

### Ce-C-Ala_c_ targets the D-loop of tRNA^Ala^ in a sequence-specific manner

**Figure 3:**
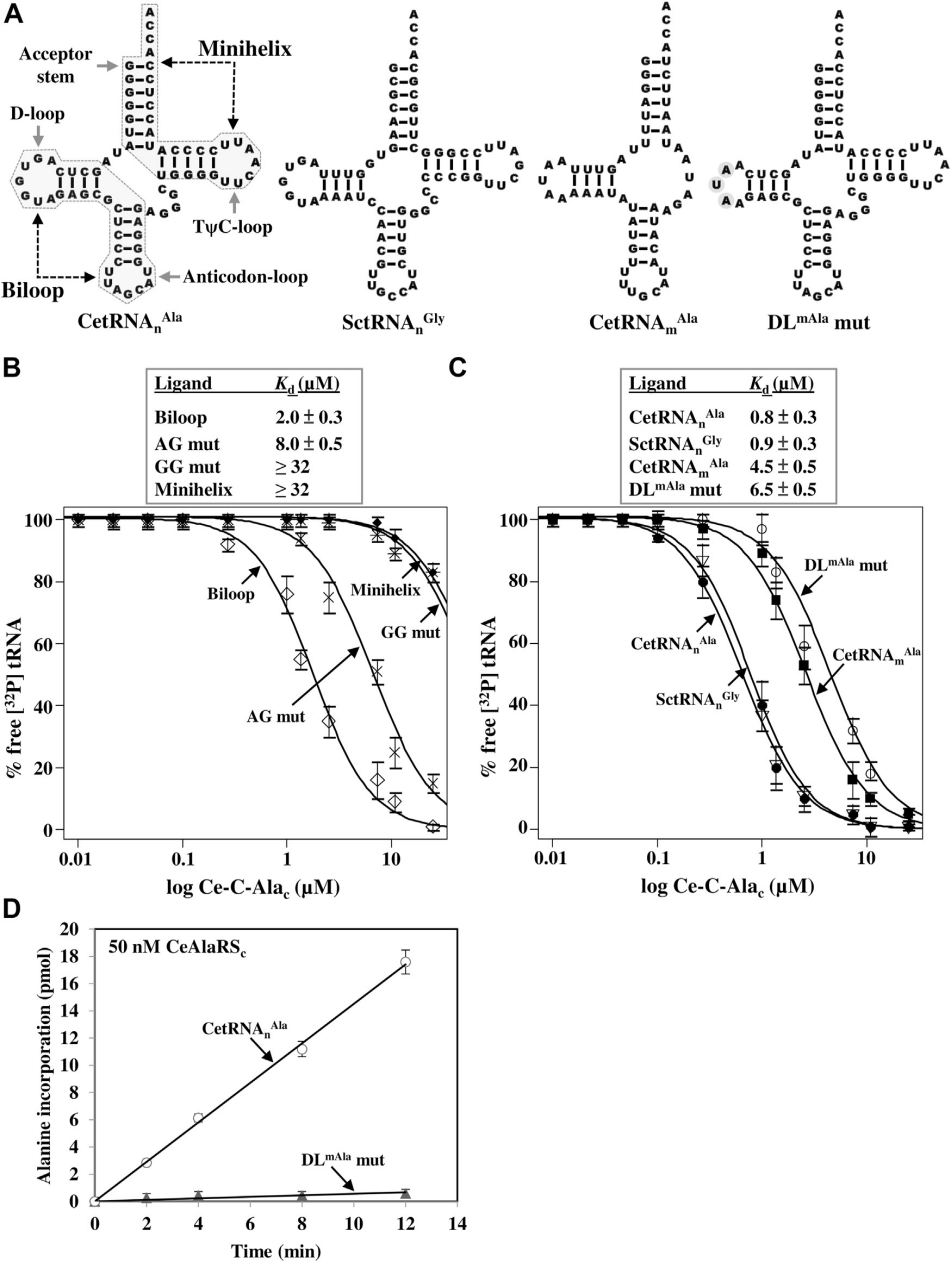
**Recognition of the D-loop by C-Ala.***A*, secondary structures of tRNAs. The minihelix (T-arm–acceptor stem) and biloop (D-arm–anticodon arm) used in this study are highlighted in *gray*. *DL*^*mAla*^*mut* denotes a CetRNA_n_^Ala^ mutant containing the D-loop of CetRNA_m_^Ala^. *B*, binding of the minihelix and biloop by Ce-C-Ala_c_. The binding affinities of Ce-C-Ala_c_ for the minihelix, biloop, and biloop mutants were determined by an EMSA with protein concentrations ranging from 32 to 0.125 μM. *C*, binding of various tRNAs by Ce-C-Ala_c_. *D*, aminoacylation of tRNAs by CeAlaRS_c_. C-Ala, C-terminal domain of alanyl-tRNA synthetase; Ce-C-Ala_c_, *Caenorhabditis elegans* cytoplasmic C-Ala; CetRNA_m_^Ala^, *C. elegans* mitochondrial tRNA^Ala^; CetRNA_n_^Ala^, *C. elegans* cytoplasmic nuclear-encoded tRNA^Ala^.

To map which part of CetRNA_n_^Ala^ is actually recognized by Ce-C-Ala_c_, we split this tRNA into two halves, one containing the acceptor stem and T-arm (termed *minihelix*) and the other containing the D-arm and anticodon arm (termed *biloop*) (Fig. 3*A*). Then, we tested these two ligands for their binding to C-Ala. Figure 3*B* shows that Ce-C-Ala_c_ robustly bound the biloop but not the minihelix (with *K*_*d*_ values of 2.0 for the biloop and >32 μM for the minihelix), suggesting that the target site resides in the biloop. Similar to the full-length Ce-C-Ala_c_, the globular subdomain also preferred the biloop over the minihelix (with *K*_*d*_ values of 2.0 for the biloop and 9.0 μM for the minihelix) (Fig. S4). Moreover, the globular subdomain bound the biloop with a higher affinity than did the helical subdomain (with *K*_*d*_ values of 2.0 for the globular subdomain and 6.0 μM for the helical subdomain).

Because Ce-C-Ala_c_ can bind various tRNAs with appreciable affinity (9), the D-loop, which is more conserved among different tRNAs than the anticodon loop, is hypothesized to be the target site. To gain further insight, we mutated the conserved invariant bases A^14^G^15^ to CC (termed *AG mutant*) or G^18^G^19^ to CC (called *GG mutant*) in the D-loop and then determined these biloop mutants’ binding affinity. As shown in Figure 3*B*, mutation at A^14^G^15^ reduced the binding affinity by four times, whereas mutation at G^18^G^19^ reduced the binding affinity by over 16 times. Thus, the D-loop is indeed the target site, and the conserved invariant bases inside, particularly G^18^G^19^, play a leading role in recognition. To ascertain whether the effect of the mutations is due to direct loss of interactions or structural changes, the secondary structures of WT and mutant biloops were monitored by CD spectroscopy. The results showed that WT and mutant biloops retain similar spectra (Fig. S5), suggesting that these mutations do not disrupt the secondary structure of the biloop.

Seeing that the D-loop is the target site, we next tried full-length tRNAs that carry a D-loop sequence identical to or very different from that of CetRNA_n_^Ala^ to gain a deeper insight into the recognition. For this purpose, CetRNA_m_^Ala^ (which lacks most of the conserved invariant bases, such as G^15^, G^18^, and G^19^, in its D-loop) and SctRNA_n_^Gly^ (which possesses a D-loop sequence identical to that of CetRNA_n_^Ala^) were chosen as ligands for the EMSA. As expected, Ce-C-Ala_c_ bound CetRNA_m_^Ala^ with an affinity approximately six times lower than that for CetRNA_n_^Ala^ (with *K*_*d*_ values of 4.5 μM for CetRNA_m_^Ala^ and 0.8 μM for CetRNA_n_^Ala^) (Fig. 3*C*). To our surprise, Ce-C-Ala_c_ bound SctRNA_n_^Gly^ with an affinity comparable to that for its cognate tRNA (with a *K*_*d*_ value of 0.9 μM). Replacing the D-loop of CetRNA_n_^Ala^ with the D-loop of its mitochondrial counterpart, yielding the DL^mAla^ mutant, resulted in an eightfold reduction in its binding affinity (with a *K*_*d*_ value of 6.5 μM). These data support our earlier finding that Ce-C-Ala_c_ targets the D-loop in a sequence-specific manner (Fig. 3, *B* and *C*). An aminoacylation assay showed that CeAlaRS_c_ strongly preferred CetRNA_n_^Ala^ over its DL^mAla^ mutant (with a difference of approximately 35 times in the aminoacylation activities) (Fig. 3*D*), laying further emphasis on the targeting specificity of C-Ala for the D-loop.

**Figure 4:**
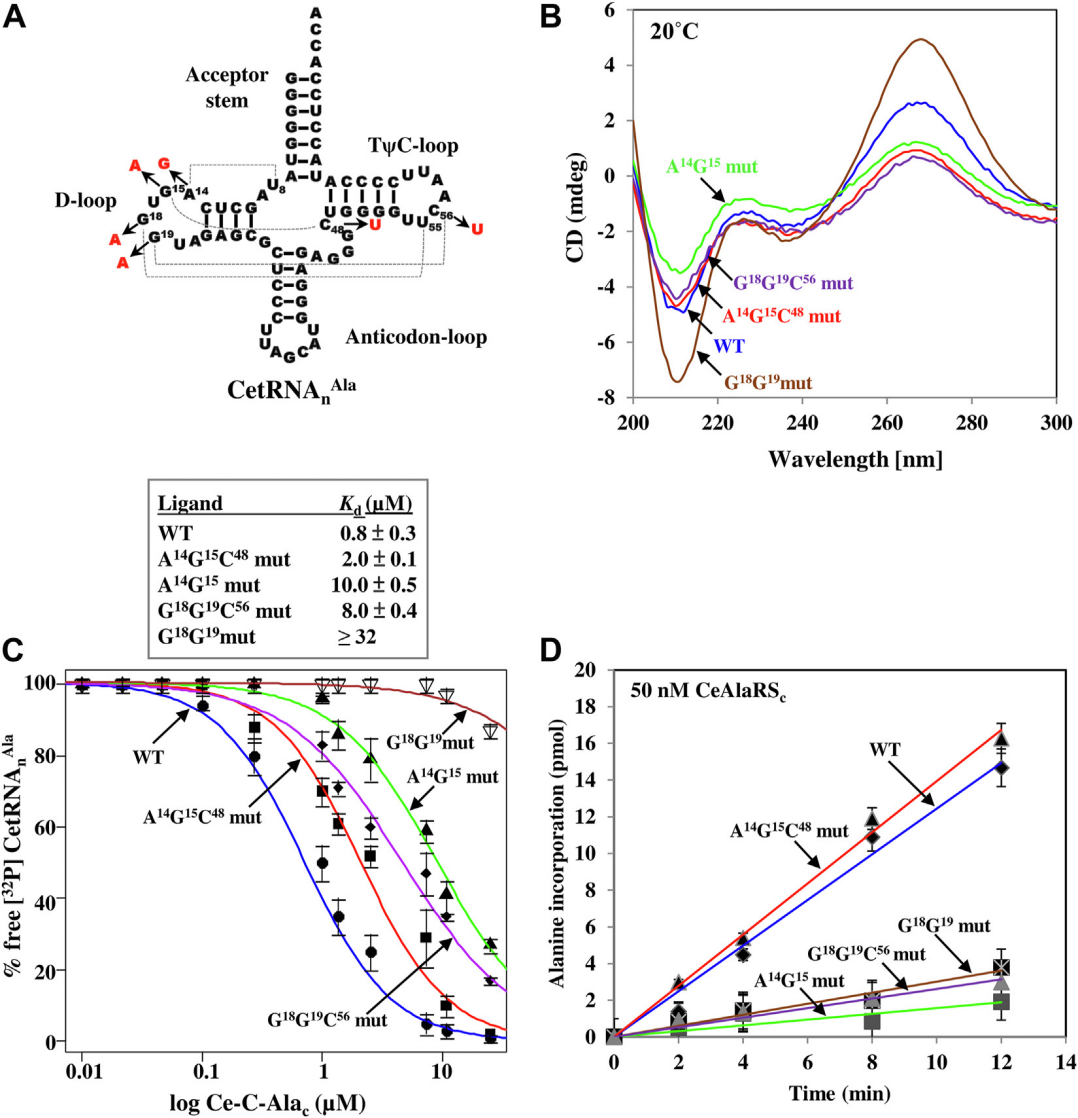
**Recognition of the L-shaped tRNA**^**Ala**^**by C-Ala.***A*, mutagenesis of the conserved invariant bases at the D-, TΨC-, and variable loops of CetRNA_n_^Ala^. For clarity, mutations are labeled in *red*. *B*, CD spectroscopic analysis. *C*, EMSA. *D*, aminoacylation assay for CetRNA_n_^Ala^ variants. C-Ala, C-terminal domain of alanyl-tRNA synthetase; CetRNA_n_^Ala^, *C. elegans* cytoplasmic tRNA^Ala^.

Because the conserved invariant bases in the D-loop are involved in the elbow formation (by the contributions of base pairs A^14^:U^8^, G^15^:C^48^, G^18^:U^55^, and G^19^:C^56^), we wondered whether the L-shaped structure also contributes to the recognition. To this end, we created several mutations in the D- and TΨC-loops of full-length CetRNA_n_^Ala^ (Fig. 4*A*), such as A^14^G^15^ to GA (termed as *A*^*14*^*G*^*15*^ *mutant*), G^18^G^19^ to AA (termed as *G*^*18*^*G*^*19*^ *mutant*), A^14^G^15^C^48^ to GAU (termed as *A*^*14*^*G*^*15*^*C*^*48*^ *mutant*, which restores the base pairing), and G^18^G^19^C^56^ to AAU (termed as *G*^*18*^*G*^*19*^*C*^*56*^ *mutant*, which restores the base pairing). We first checked by CD spectroscopy whether these mutations disrupt or maintain the L-shaped structure of tRNA^Ala^. As the tRNA conformation is an A-form double helix, it has a distinctive negative peak at 208 nm (16). As shown in Figure 4*B*, mutation at A^14^G^15^ distinctly decreased the negative peak at 208 nm (see *A*^*14*^*G*^*15*^ *mutant*), suggesting that the tRNA is denatured by the mutation. However, when a complementary mutation was introduced, the tRNA conformation was restored (see *A*^*14*^*G*^*15*^*C*^*48*^ *mutant*). Interestingly, mutation at G^18^G^19^ increased, rather than decreased, the negative peak at 208 nm (see *G*^*18*^*G*^*19*^ *mutant*), suggesting that this mutation does not denature the tRNA. Not surprisingly, *G*^*18*^*G*^*19*^*C*^*56*^ *mutant* (with a complementary mutation) maintained a near-WT conformation. Thus, A^14^G^15^ plays a role much more important than G^18^G^19^ in stabilizing the elbow structure of the L-shaped tRNA, a scenario consistent with previous reports (17, 18).

To examine whether the elbow structure also plays a role in tRNA recognition by Ce-C-Ala_c_, the aforementioned tRNA^Ala^ variants were tested in an EMSA. As shown in Figure 4*C*, mutation of A^14^G^15^ to GA (which disrupted the tRNA conformation) reduced the binding affinity by ∼12-fold (with its *K*_*d*_ value changed from 0.8 to 10 μM). However, the binding affinity was partially restored (with a *K*_*d*_ value of 2 μM) when a complementary mutation was introduced (see *A*^*14*^*G*^*15*^*C*^*48*^ *mutant*), suggesting that the elbow structure is crucial for tRNA^Ala^ recognition and the sequence A^14^G^15^ plays only a relatively minor role in this regard. Most interestingly, mutation of G^18^G^19^ to AA (which still preserved the tRNA conformation) reduced the binding affinity by more than 40-fold (with a *K*_*d*_ value of ˃32 μM), suggesting that the sequence G^18^G^19^ plays a leading role in tRNA recognition. Introduction of a complementary mutation into the *G*^*18*^*G*^*19*^ *mutant* (see *G*^*18*^*G*^*19*^*C*^*56*^ *mutant*) only slightly restored its binding affinity (with a *K*_*d*_ value of 8 μM), laying further emphasis of the importance of the sequence G^18^G^19^. Altogether, our data suggest that both the invariant bases G^18^G^19^ and elbow structure of tRNA are important for tRNA^Ala^ recognition by C-Ala, with G^18^G^19^ being the major determinant.

We next checked whether these tRNA mutants can be efficiently charged by CeAlaRS_c_. As shown in Figure 4*D*, mutation at A^14^G^15^ of CetRNA_n_^Ala^ drastically reduced its aminoacylation by AlaRS (see *A*^*14*^*G*^*15*^ *mutant*). As expected, the aminoacylation activity was fully restored when a complementary mutation was introduced (see *A*^*14*^*G*^*15*^*C*^*48*^ *mutant*), suggesting that the elbow structure indeed plays an important role in tRNA^Ala^ recognition and aminoacylation. Despite the fact that mutation at G^18^G^19^ did not disrupt the tRNA conformation, this mutation drastically reduced the aminoacylation activity. Moreover, a complementary mutation (see *G*^*18*^*G*^*19*^*C*^*56*^ *mutant*) failed to restore its aminoacylation activity, laying further emphasis on the importance of G^18^G^19^ in recognition.

**Figure 5:**
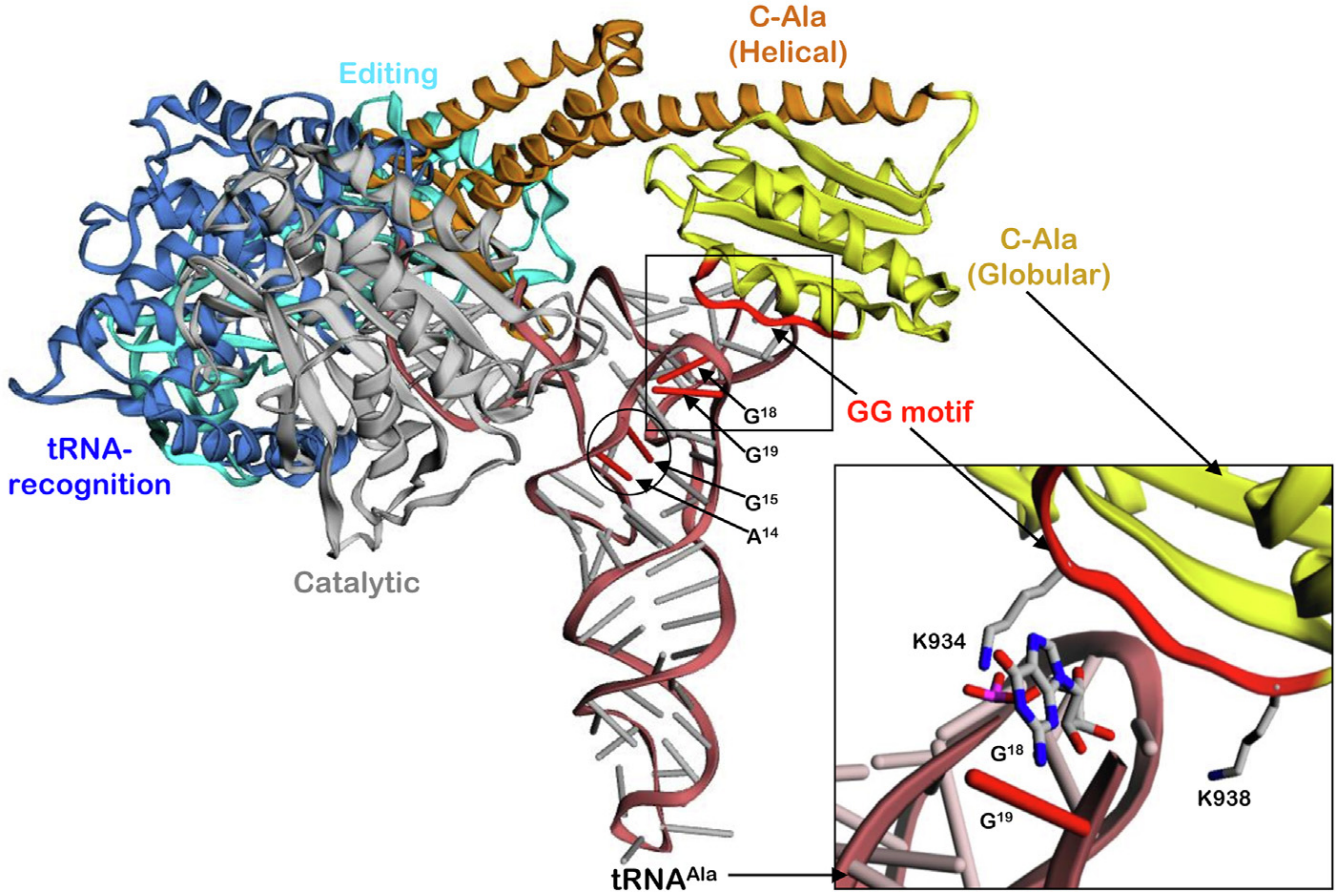
**A docking model for the CeAlaRS**_**c**_**–tRNA**^**Ala**^**complex.** The conserved invariant bases (A^14^, G^15^, G^18^, and G^19^) in the D-loop of tRNA^Ala^ and the lysine residues flanking the GG-motif (K934 and K938) in C-Ala are highlighted for clarity. K934 makes a direct contact with G^18^, whereas K938 seems to have no direct contact with any of the invariant bases. CeAlaRS_c_, *Caenorhabditis elegans* cytoplasmic alanyl-tRNA synthetase.

**Figure 6:**
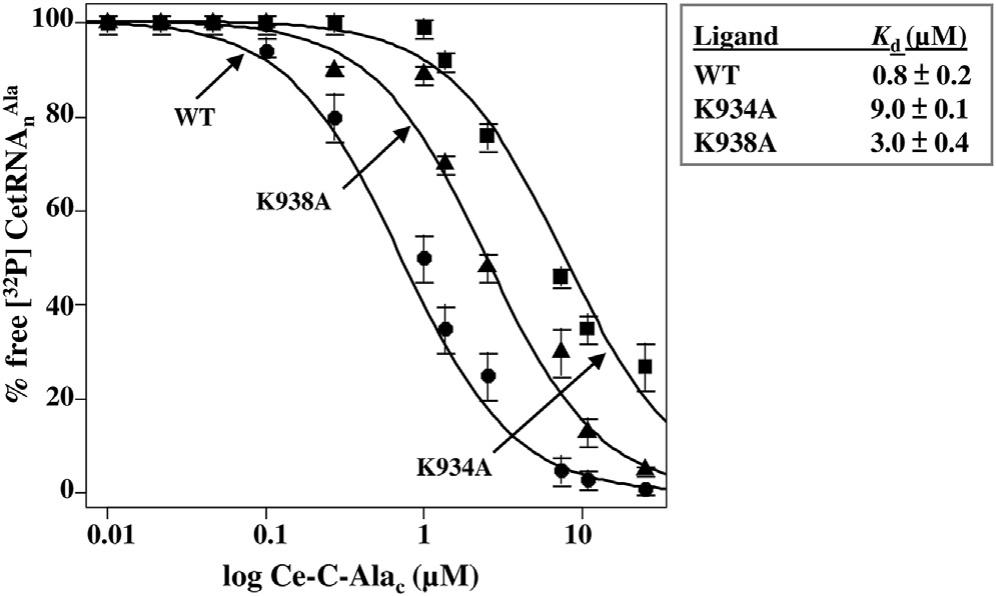
**tRNA binding by Ce-C-Ala**_**c**_**mutants.** The binding affinities of Ce-C-Ala_c_ mutants (K934A and K938A) for CetRNA_n_^Ala^ were determined by an EMSA with protein concentrations ranging from 32 to 0.125 μM. Ce-C-Ala_c_, *Caenorhabditis elegans* cytoplasmic C-Ala; CetRNA_n_^Ala^, *C. elegans* cytoplasmic tRNA^Ala^.

To gain a deeper insight, molecular docking was performed for CeAlaRS_c_ and tRNA^Ala^ (Fig. 5) using HADDOCK 2.4 (https://wenmr.science.uu.nl/haddock2.4/) (19, 20)). The docking model showed that C-Ala contacts the invariant base G^18^ in the D-loop through K934 in its GG-motif. To verify this model, we introduced mutations into the lysine residues (K934A and K938A) surrounding the GG-motif and tested the resultant mutants for their tRNA-binding affinities. Consistent with the docking model, mutation at K934A reduced the binding affinity of Ce-C-Ala_c_ for tRNA^Ala^ by 11-fold. Although K938A seems to have no direct contact with any of the conserved invariant bases in the D-loop, mutation at this lysine residue reduced the binding affinity by threefold (Fig. 6).

### Ce-C-Ala_m_ also plays an important role in tRNA binding and aminoacylation

**Figure 7:**
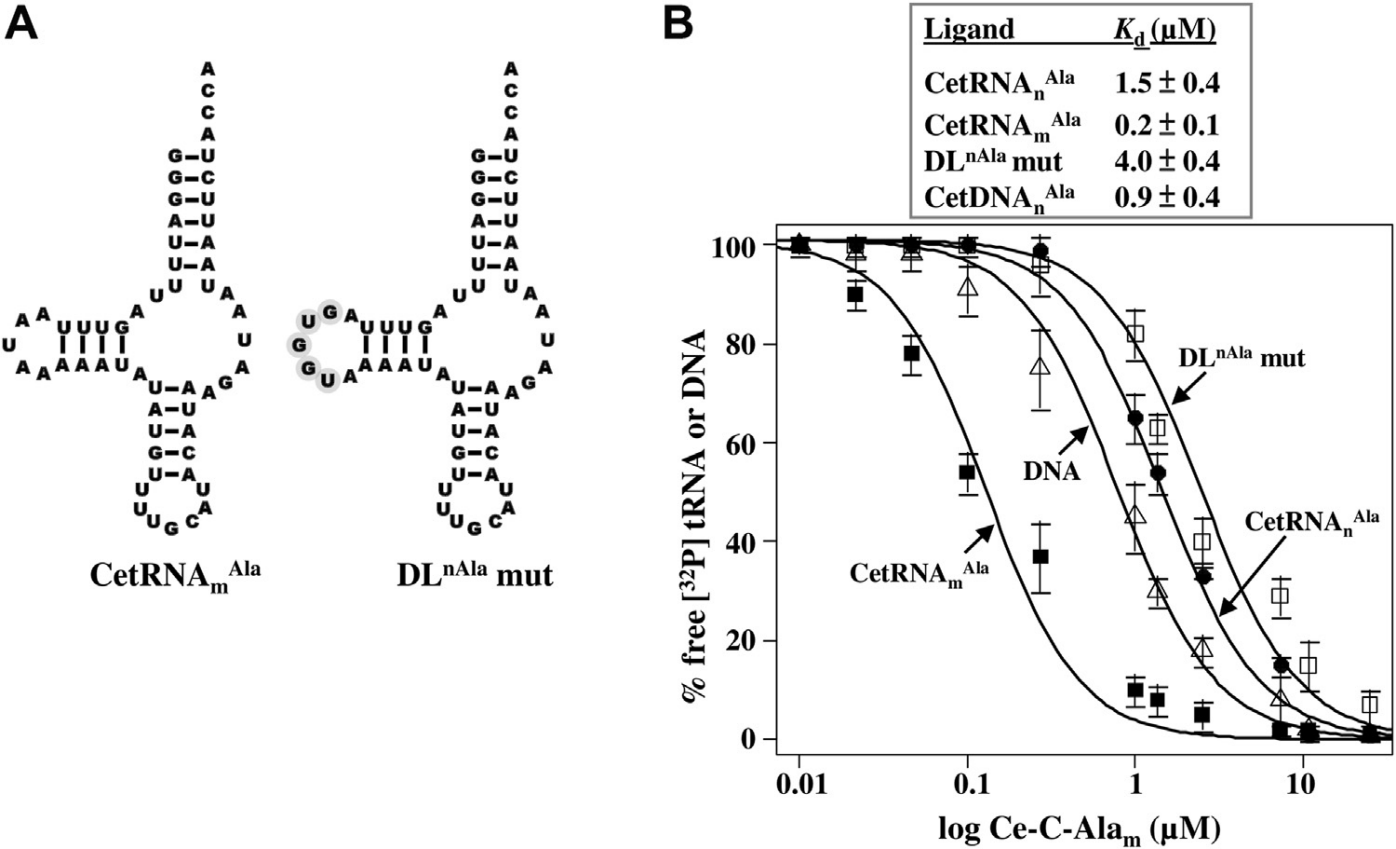
**Ce-C-Ala**_**m**_**targets the D-loop of its cognate tRNA.***A*, secondary structures of tRNAs. *DL*^*nAla*^*mut* denotes a CetRNA_m_^Ala^ mutant containing the D-loop of CetRNA_n_^Ala^. *B*, the binding affinities of Ce-C-Ala_m_ for DNA and various tRNAs were determined by an EMSA with protein concentrations ranging from 32 to 0.125 μM. Ce-C-Ala_m_, *Caenorhabditis elegans* mitochondrial C-Ala.

Because C-Ala is the major tRNA-binding module for *E. coli* AlaRS (7), deletion of this domain drastically impairs the enzyme’s aminoacylation activity (8). Likewise, deletion of C-Ala from CeAlaRS_c_ drastically impairs its aminoacylation activity (9). This prompted us to ask whether the residual C-Ala of CeAlaRS_m_ plays a role in aminoacylation and, if so, whether it is specific to the D-loop of its cognate tRNA. To this end, we purified C-Ala of CeAlaRS_m_ (Ce-C-Ala_m_) and tested it for its tRNA binding. Although the D-loop of CetRNA_m_^Ala^ lacks most of the conserved invariant bases (Fig. 7*A*), Ce-C-Ala_m_ robustly bound this tRNA (with a *K*_*d*_ value of 0.2 μM) (Fig. 7*B*). Replacing the D-loop of CetRNA_m_^Ala^ with that of its cytoplasmic counterpart, which yielded the DL^nAla^ mutant, caused a 20-fold reduction in its binding affinity (with a *K*_*d*_ value of 4 μM). In addition, Ce-C-Ala_m_ bound CetRNA_n_^Ala^ with a 7.5 times lower affinity. This result suggests that Ce-C-Ala_m_ specifically targets the D-loop of its cognate tRNA. Remarkably, this relatively short domain also robustly bound DNA (with a *K*_*d*_ value of 0.9 μM). In view of the fact that Ce-C-Ala_m_ retains only one-fourth the size of its cytoplasmic counterpart, the observation that it robustly binds both DNA and tRNA is surprising.

**Figure 8:**
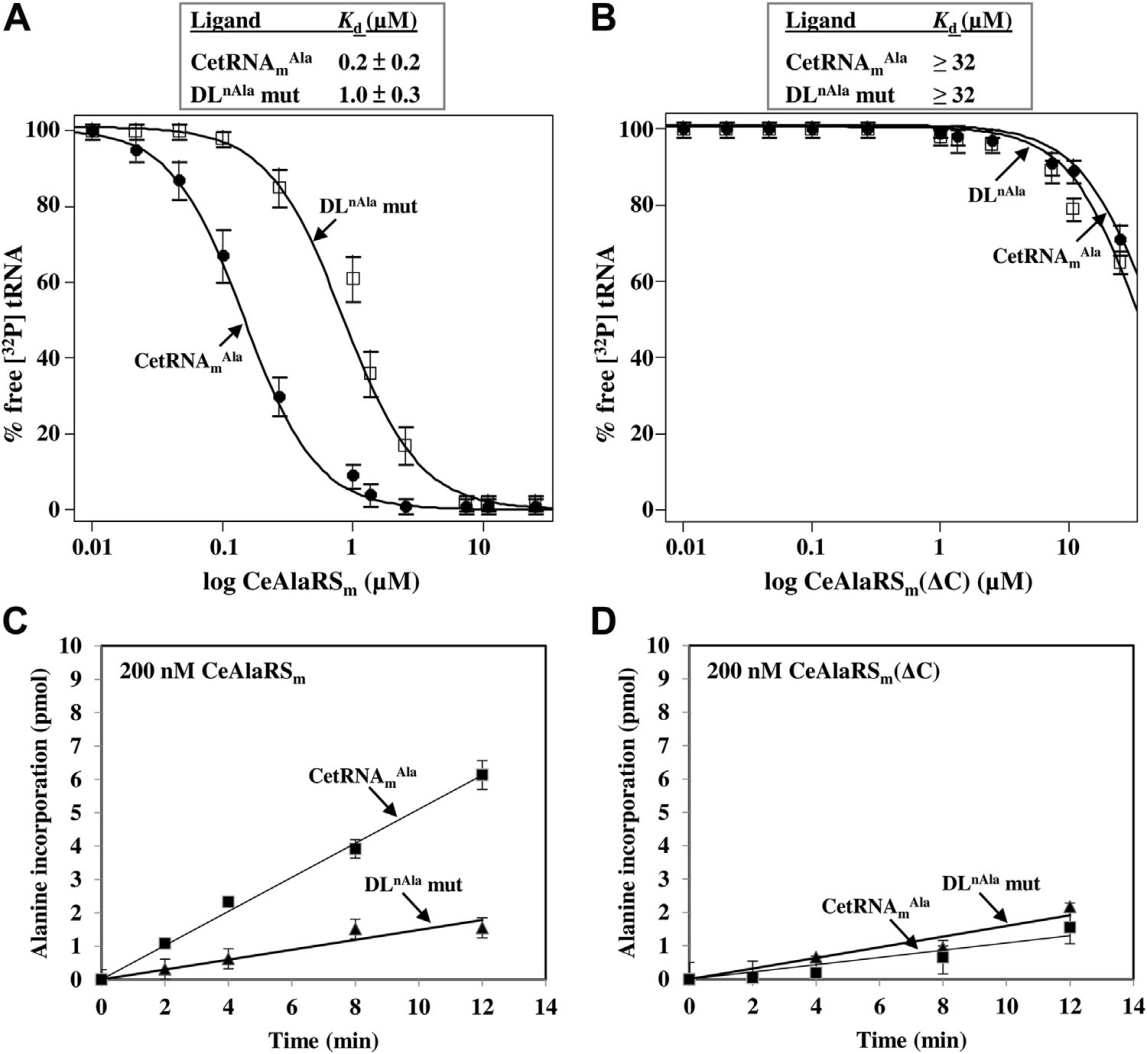
**Ce-C-Ala**_**m**_**plays an important role in tRNA binding and aminoacylation.** The binding affinities of (*A*) CeAlaRS_m_ and (*B*) its C-Ala deletion mutant for tRNAs were determined by an EMSA with protein concentrations ranging from 32 to 0.125 μM. The aminoacylation activities of (*C*) CeAlaRS_m_ and (*D*) its C-Ala deletion mutant were determined by testing their ability to incorporate ^3^H-alanine into CetRNA_m_^Ala^ and its D-loop mutant. Ce-C-Ala_m_, *Caenorhabditis elegans* mitochondrial C-Ala.

To explore whether this short C-Ala indeed plays a role in tRNA binding, we next compared the tRNA-binding affinities of the full-length CeAlaRS_m_ and its C-Ala deletion mutant. Figure 8, *A* and *B* shows that CeAlaRS_m_ bound CetRNA_m_^Ala^ with an affinity approximately five times higher than that for its DL^nAla^ mutant (with *K*_*d*_ values of 0.2 μM for CetRNA_m_^Ala^ and 1.0 μM for the DL^nAla^ mutant), suggesting that the D-loop sequence plays a role in tRNA recognition. In contrast, CeAlaRS_m_(ΔC) failed to bind either ligand with an appreciable affinity (with *K*_*d*_ values of greater than 32 μM for both), suggesting that Ce-C-Ala_m_ is the major tRNA-binding module for CeAlaRS_m_. We next tested the aminoacylation activities of CeAlaRS_m_ and its C-Ala deletion mutant. As shown in Figure 8, *C* and *D*, deleting C-Ala from CeAlaRS_m_ significantly reduced its aminoacylation activity (by 3.6 times) toward CetRNA_m_^Ala^. Whereas CeAlaRS_m_ distinctly preferred CetRNA_m_^Ala^ over its DL^nAla^ mutant (with a 3.4-fold difference in aminoacylation activities) (Fig. 8*C*), CeAlaRS_m_(ΔC) failed to distinguish the CetRNA_m_^Ala^ from its DL mutant (Fig. 8*D*).

We next determined the kinetic parameters for aminoacylation of CetRNA_m_^Ala^ by CeAlaRS_m_ and its C-Ala deletion mutant. As shown in Table 1, replacing the D-loop of CetRNA_m_^Ala^ with that of its cytoplasmic counterpart had little effect on the *k*_cat_ value of CeAlaRS_m_, but this replacement increased the enzyme’s *K*_*M*_ value for CetRNA_m_^Ala^ by up to 3.8-fold. This result suggests that the D-loop sequence plays an important role in tRNA binding by the enzyme. As expected, deletion of C-Ala from CeAlaRS_m_ had only a minor effect on the enzyme’s *k*_cat_ value (twofold). However, this deletion distinctly increased the enzyme’s *K*_*M*_ value (4.5-fold) for CetRNA_m_^Ala^. Altogether, these results suggest that the residual C-Ala domain of CeAlaRS_m_ also acts as the major tRNA-binding module and preferentially recognizes the D-loop of its cognate tRNA. Thus, Ce-C-Ala_m_, no matter how improbable, functions as a D-loop-specific tRNA-binding domain.

**Table 1:** Kinetic parameters for aminoacylation of CetRNA_m_^Ala^ by CeAlaRS_m_ and its C-Ala deletion mutant

Enzyme	tRNA	*k*_cat_ (*×*10^−3^ s^−1^)	*K*_*M*_ (×10^−3^ μM)	*k*_cat_/*K*_*M*_ (×10^−3^ μM^−1^ s^−1^)
CeAlaRS_m_	CetRNA_m_^Ala^	1.0 ± 0.3	222.3 ± 20.3	4.5 ± 1.3
	DL^nAla^ mut	1.1 ± 0.5	851.7 ± 12.5	1.3 ± 0.3
CeAlaRS_m_(ΔC)	CetRNA_m_^Ala^	0.5 ± 0.05	1009.2 ± 22.7	0.5 ± 0.05
	DL^nAla^ mut	0.4 ± 0.03	935.0 ± 83	0.4 ± 0.08

## Discussion

### *C. elegans* C-Ala plays an important role in tRNA binding and aminoacylation

Despite AlaRS retaining a conserved structure, its C-terminal domain (C-Ala) is highly diverged during evolution (7, 8) (Figs. 1 and S1). Paradoxically, removal of C-Ala from *E. coli* or *A. fulgidus* AlaRS drastically reduces its aminoacylation activity (7, 8, 21), but removal of C-Ala from human cytoplasmic AlaRS has little effect on its aminoacylation activity (8). Human cytoplasmic C-Ala, while losing the canonical tRNA-binding activity, is able to enter the nucleus and to bind DNA (8). A recent study further showed that this domain can be expressed as a freestanding protein and play a role in cytoprotection, inflammatory response, and cell differentiation (22). Although the predicted structure of Ce-C-Ala_c_ is highly similar to that of human cytoplasmic C-Ala, deletion of C-Ala from CeAlaRS_c_ severely impairs its aminoacylation activity (9), suggesting that this domain still plays an important role in aminoacylation. Most surprisingly, although Ce-C-Ala_m_ retains negligible sequence similarity to any known C-Ala domains (Fig. 1), it maintains targeting specificity to the D-loop of its cognate tRNA (Fig. 7*B*). Hence, deletion of the short C-Ala from CeAlaRS_m_ distinctly impairs its tRNA-binding and aminoacylation activities (Fig. 8), suggesting that Ce-C-Ala_m_ still functions as a tRNA-binding domain.

*E. coli* C-Ala folds into a homodimer with a parallel organization (23) and strongly prefers tRNA^Ala^ over DNA (9). In contrast, human cytoplasmic C-Ala folds into a monomer under reducing conditions and a homodimer with an antiparallel organization under oxidizing conditions (8). Interestingly, both forms robustly bind DNA (8, 9). Despite possessing a predicted tertiary structure resembling that of human cytoplasmic C-Ala, Ce-C-Ala_c_ folds only into a monomer (9), possibly because of the lack of the equivalent cysteine residues required for dimerization of human cytoplasmic C-Ala (8). However, different from all known C-Ala domains, Ce-C-Ala_c_ robustly binds both ligands. It thus appears that the helical subdomain of Ce-C-Ala_c_ has been repurposed from mediating dimerization to DNA binding, whereas the globular subdomain has retained tRNA-binding activity. In contrast, Ce-C-Ala_m_, which shares little sequence similarity to Ce-C-Ala_c_, forms a predicted structure resembling the N-terminal part of the helical subdomain of Ce-C-Ala_c_ (Fig. 1*C*). Unexpectedly, this helical structure binds tRNA as well as DNA (Fig. 7). It will be interesting to further explore the biological function of this DNA-binding property. One possibility is that Ce-C-Ala_c_ binds the promoter of the CeAlaRS gene and regulates its transcription. A scenario of this type has previously been reported in *E. coli* AlaRS. This enzyme represses transcription of its own gene by binding specifically to a palindromic sequence flanking the gene’s transcription start site (24).

### D-loop sequence of tRNA^Ala^ is important for recognition by *C. elegans* C-Ala

Our previous study showed that Ce-C-Ala_c_ retains a relaxed tRNA specificity, albeit with a distinct preference for its cognate tRNA. Most interestingly, this domain strongly prefers the L-shaped tRNA (9). We showed herein that Ce-C-Ala_c_ mainly targets the conserved invariant bases in the D-loop of tRNA^Ala^ (Figure 3, Figure 4, Figure 5, Figure 6), which might explain why this domain can bind various tRNAs with appreciable affinity (9). Because the conserved invariant bases account for more than half of the bases in the D-loop (Fig. S6), it is unsurprising to find that some unrelated tRNAs possess similar, or even identical, D-loop sequences. For example, four nematode tRNAs (CetRNA_n_^Ala^, CetRNA_n_^Gly^, CetRNA_n_^Thr^, and CetRNA_n_^Pro^) and four yeast tRNAs (SctRNA_n_^Gly^, SctRNA_n_^Thr^, SctRNA_n_^Pro^, and SctRNA_n_^Cys^) possess an identical D-loop sequence—AGUGGUA (Fig. S6). Although *E. coli* C-Ala shares only 30% similarity with Ce-C-Ala_c_ (Figs. 1*B* and S1), it also targets the D-loop of tRNA^Ala^ (7, 8) and exhibits a relaxed tRNA specificity (9), lending support to the hypothesis that it also recognizes the conserved invariant bases in the D-loop. Paradoxically, however, human mitochondrial C-Ala targets the variable loop, instead of the D-loop, of its cognate tRNA (3). Despite the fact that human cytoplasmic C-Ala possesses a tertiary structure resembling that of Ce-C-Ala_c_, it poorly binds tRNA (9). Most interestingly, although Ce-C-Ala_m_ bears little resemblance to its cytoplasmic counterpart, it maintains targeting specificity to the D-loop of its cognate tRNA (Fig. 7*B*). In addition to C-Ala, a number of proteins and domains have been shown to bind the elbow region of tRNA, including the C domain of LeuRS (which interacts with the T-loop and variable arm) (25), the N domain of ArgRS (which recognizes the base A20 in the D-loop) (26), Trbp111 (which is specific for the L-shaped structure) (27), the C domain of Arc1p (which interacts with the T-arm) (28), the PPR domain of PRORP (which accommodates the G19:C56 base pair in the elbow) (29), and many others (30). However, unlike C-Ala of AlaRS, these elbow-binding domains are not linked to both a synthetase and a free-standing editing domain. Representative EMSA figures are shown in Fig. S7.

Three types of free-standing AlaXps (Ia, Ib, and II) are found to act in *trans* to hydrolyze mischarged tRNA^Ala^ (31), but only type II AlaXp contains the C-Ala domain. A phylogenetic analysis suggests that AlaXp-II is derived from AlaXp-I and that an ancient AlaRS may have acquired the free-standing AlaXp-II by fusion (1). As C-Ala functions in bringing together aminoacylation and editing centers on one tRNA, it may have played a critical role in the evolution of AlaRSs by coupling aminoacylation to editing to prevent mistranslation (7). Perhaps for this reason, the residual C-Ala of *C. elegans* mitochondrial AlaRS still preserves the tRNA-binding activity.

### D-loop recognition occurs primarily through C-Ala’s affinity, but not specificity, for its cognate tRNA

The G3:U70 base pair in the acceptor stem is the primary identity element of tRNA^Ala^ in organisms ranging from *E. coli* to human cytoplasm. Although D-loop recognition also contributes to tRNA specificity (9), the major tRNA specificity arises from selection of the G3:U70 base pair (10). Therefore, D-loop recognition is achieved primarily through C-Ala’s affinity for its cognate tRNA and not *via* selectivity against noncognate tRNAs.

### Coevolution of AlaRS and tRNA^Ala^

It is believed that as an ancient AlaRS (containing a catalytic domain and a tRNA-recognition domain) acquired an editing domain and C-Ala to yield the four-domain structure, an ancient tRNA^Ala^ (containing an acceptor stem plus a TψC-arm) acquired a biloop (containing a D-arm and an anticodon arm) to form the L-shaped structure (32). Phylogenetic analysis demonstrated that the existing *C. elegans* cytoplasmic and mitochondrial homologs of AlaRS were closer to the bacterial group than to the archaeal group. Thus, it is likely that these two homologs were descended from duplication of a mitochondrion-type predecessor (Fig. S3) (9). However, the phylogenetic tree based only on C-Ala showed that the eukaryotic C-Ala domains, including the nematode C-Ala homologs, are not closer to the bacterial C-Ala domains (Fig. S2). Despite of that, both *C. elegans* C-Ala domains still retain a tRNA-binding activity. It is therefore likely that the residual C-Ala of CeAlaRS_m_ resulted from secondary loss of an intact C-Ala during evolution (9). As for its interacting partner, the D-loop, certain bases in this loop are compelled to remain invariant to pair with certain bases in the TψC-loop to form the elbow of the L-shaped tRNA (30). However, losing the T-loop, as in the scenario of CetRNA_m_^Ala^, lifted the selection pressure off the D-loop, giving rise to its wild deviation from the norm (Fig. 3*A*). Despite all this, the residual C-Ala of CeAlaRS_m_ is still specific to the D-loop of its cognate tRNA (Figs. 7 and 8). Regardless of the detailed interpretation, this study highlights the underlying mechanism of how C-Ala targets the D-loop of tRNA^Ala^.

## Experimental procedures

### Construction of plasmids

Cloning of the open reading frames encoding CeAlaRS_c_ and CeAlaRS_m_ was described earlier (9). Cloning of the DNA sequence encoding Ce-C-Ala_c_ into pET21b (an *E. coli* expression vector with a T7 promoter followed by multiple cloning sites and a short sequence encoding a His_6_ tag) followed a similar strategy. In brief, a set of gene-specific primers was designed to amplify the target sequence *via* a PCR using the plasmid encoding CeAlaRS_c_ as a template. The forward primer with an NdeI site was annealed to the 5′-end sequence of Ce-C-Ala_c_, whereas the reverse primer with an XhoI site was annealed to the 3′-end sequence immediately upstream of the stop codon. The PCR-amplified DNA fragment (bp +2269 to +2904) was cloned into the NdeI–XhoI sites of pET21b following treatment with NdeI and XhoI. Cloning of the genes encoding CeAlaRS_m_(ΔC) (containing bp +1 to +2145), Ce-C-Ala_m_ (containing bp +2146 to +2379), and the helical (containing bp +2269 to +2574) and globular (containing bp +2575 to +2904) subdomains of Ce-C-Ala_c_ followed a similar strategy.

For protein purification, the plasmids were individually transformed into an *E. coli* strain, BL21-CodonPlus(DE3), and the transformants were induced with 1 mM isopropyl β-d-1-thiogalactopyranoside for 4 h at 30 °C. The His_6_-tagged target proteins were purified to homogeneity through nickel–nitrilotriacetic acid affinity chromatography as previously described (33).

### EMSA

Protein–RNA- or protein–DNA-binding affinities were determined by an EMSA, as described earlier (34). The RNA or DNA ligand was ^32^P-radiolabeled at the 5′-end. For each complex formation, ∼10,000 cpm of RNA (or DNA) (diluted with cold ligand to 0.1 μM final concentration) was incubated for 10 min on ice with variable concentrations of C-Ala or AlaRS in a total volume of 20 μl containing 20 mM Hepes, pH 7.5, 20 mM KCl, 5 mM MgCl_2_, and 2 mM DTT. Glycerol was added to a final concentration of 5% in each sample prior to loading on a native 5% 29:1 polyacrylamide gel. The gel was run at 100 V for 30 to 45 min at 4 °C in 0.5× Tris–borate–EDTA and then dried. The dried gel was exposed to X-ray film at −80 °C with an intensifying screen. The signal intensity was compared through ImageJ 1.53e (https://imagej.nih.gov/ij/download.html) to measure the remaining free ^32^P-RNA (or ^32^P-DNA).

### *In vitro* transcription of tRNA

Preparation of various tRNA transcripts followed a standard protocol. We cloned a DNA duplex containing a T7 promoter followed by a sequence encoding the tRNA into the SmaI site of pUC18. We enriched the transcription template through PCR amplification of the insert. We performed *in vitro* transcription at 37 °C for 3 h with 0.3 μM T7 RNA polymerase in 20 mM Tris–HCl (pH 8.0), 150 mM NaCl, 20 mM MgCl_2_, 5 mM DTT, 1 mM spermidine, and 2 mM of each NTP. We purified the transcript using a 15% denaturing urea–polyacrylamide gel. After ethanol precipitation and vacuum drying, we dissolved the RNA pellet in 1× TE buffer (20 mM Tris–HCl [pH 8.0] and 1 mM EDTA), refolded it by heating it to 80 °C for 3 min, and gradually cooled it to 55 °C before adding 2 mM of MgCl_2_. As soon as the temperature reached approximately 25 °C, we collected the refolded tRNA and stored it in a −80 °C freezer.

### Aminoacylation assay

We carried out aminoacylation at ambient temperature in a buffer containing 50 mM Hepes (pH 7.5), 50 mM KCl, 15 mM MgCl_2_, 5 mM DTT, 10 mM ATP, 0.1 mg/ml bovine serum albumin, 5 μM tRNA, 20 μM alanine (1.34 μM ^3^H-alanine; PerkinElmer), and 200 nM AlaRS. At various time points, we quenched reactions by spotting 10 μl aliquots of the reaction mixture onto Whatman filter pads (Maidstone) that had been presoaked in 5% trichloroacetic acid and 2 mM alanine. Before liquid scintillation counting, we washed the filter pads three times for 15 min each in ice-cold 5% trichloroacetic acid. We determined active protein concentrations by active site titration as previously described (35). We obtained aminoacylation data from three independent experiments and averaged them.

### CD spectroscopy

The conformations of WT and mutant biloops (at 25 μM) were evaluated using an AVIV Model 410 CD spectrometer. The CD spectrum from 200 to 300 nm was measured at 20 °C and 80 °C with a quartz cell (0.1 cm path length) at a scan speed of 50 nm/min and a bandwidth of 2 nm. The CD signal at each wavelength was collected for 5 s and averaged. The data excluding the buffer background signals were shown in the final spectra. The RNA samples were prepared in 10 mM Tris–HCl, pH 7.8 (36, 37).

## Data availability

Data supporting the findings of this study are available within the article and its supporting information.

## Supporting information

This article contains supporting information.

## Conflict of interest

The authors declare that they have no conflicts of interest with the contents of this article.
