# Supplementary material for: Sequence-specific targeting of Caenorhabditis elegans C-Ala to the D-loop of tRNAAla
Source: J Biol Chem. 2023 Aug 9;299(9):105149. doi: 10.1016/j.jbc.2023.105149 (PMC10485164; doi:10.1016/j.jbc.2023.105149)
Supplement: Supporting Figures S1–S7 [file mmc1.pdf]

	Ce-C	Hs-C	Dr-C	Sc-CM	Hs-M	Dr-M	Ec	Af
**Ce-C**		**36**	**37**	**21**	**22**	**30**	**34**	**42**
**Hs-C**			**75**	**25**	**30**	**26**	**28**	**26**
**Dr-C**				**25**	**27**	**27**	**37**	**28**
**Sc-CM**					***ND**	**25**	**40**	***ND**
**Hs-M**						**42**	***ND**	***ND**
**Dr-M**							***ND**	***ND**
**Ec**								**26**
**Af**								

*ND, not determined due to no significant similarity found.

Figure S1. Sequence similarities between C-Ala domains. Ce, *Caenorhabditis elegans*; Hs, *Homo sapiens*; Dr, *Danio rerio*; Sc, *Saccharomyces cerevisiae*; Ec, *Escherichia coli*; Af, *Archaeoglobus fulgidus*. C and M respectively denote cytoplasmic and mitochondrial.

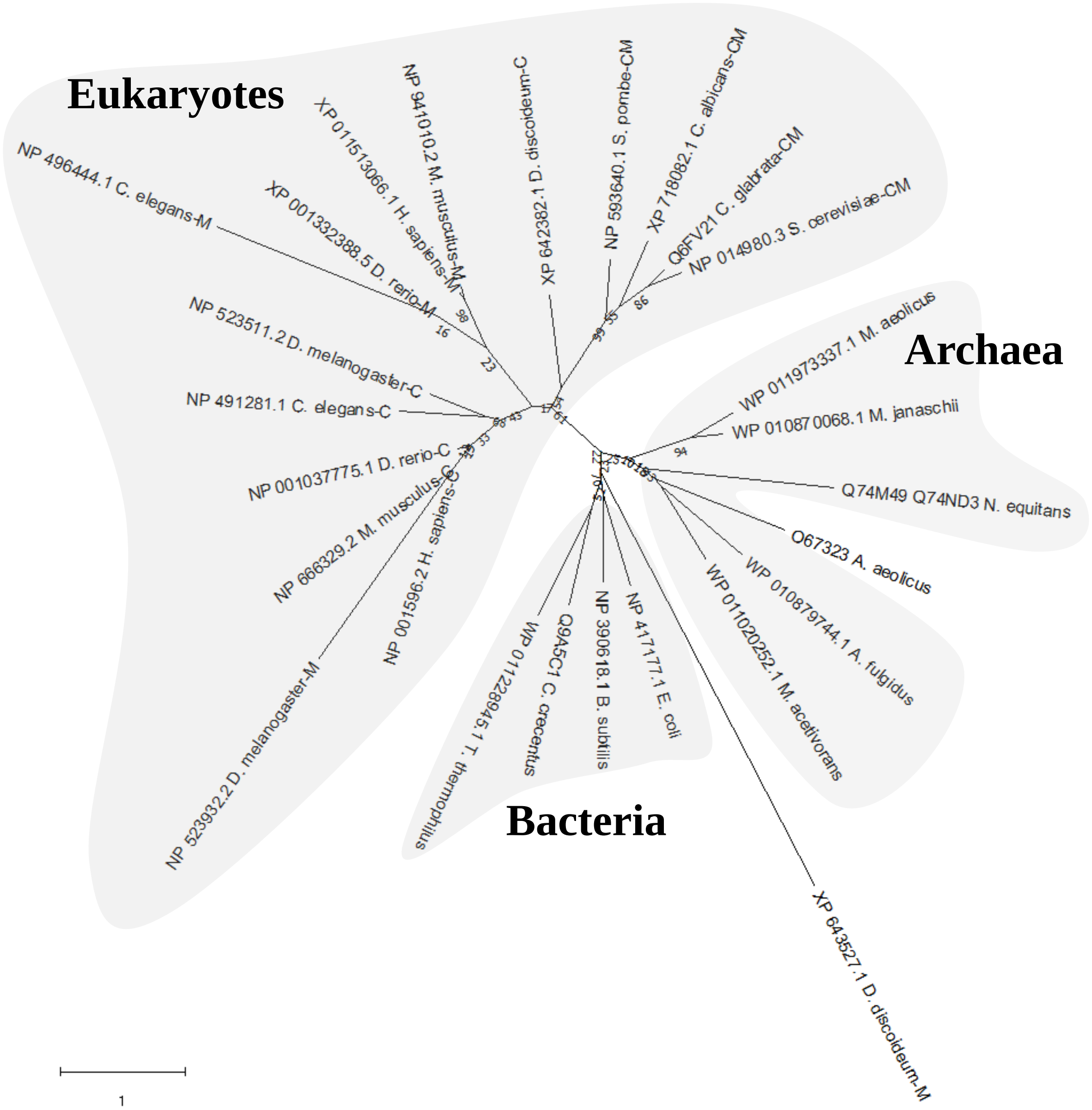

Figure S2. Evolutionary analysis of C-Ala by Maximum Likelihood method. The evolutionary history was inferred by using the Maximum Likelihood method and the Tamura–Nei model. The tree with the highest log likelihood (-1519.13) is shown. The percentage of trees in which the associated taxa clustered together is shown next to the branches. Initial tree(s) for the heuristic search were obtained automatically by applying the Neighbor–Join and BioNJ algorithms to a matrix of pairwise distances estimated using the Tamura–Nei model, and then selecting the topology with the superior log likelihood value. The tree is drawn to scale, with branch lengths measured in the number of substitutions per site. This analysis involved 30 nucleotide sequences. There was a total of 92 positions in the final data set. Evolutionary analyses were conducted in MEGA X.

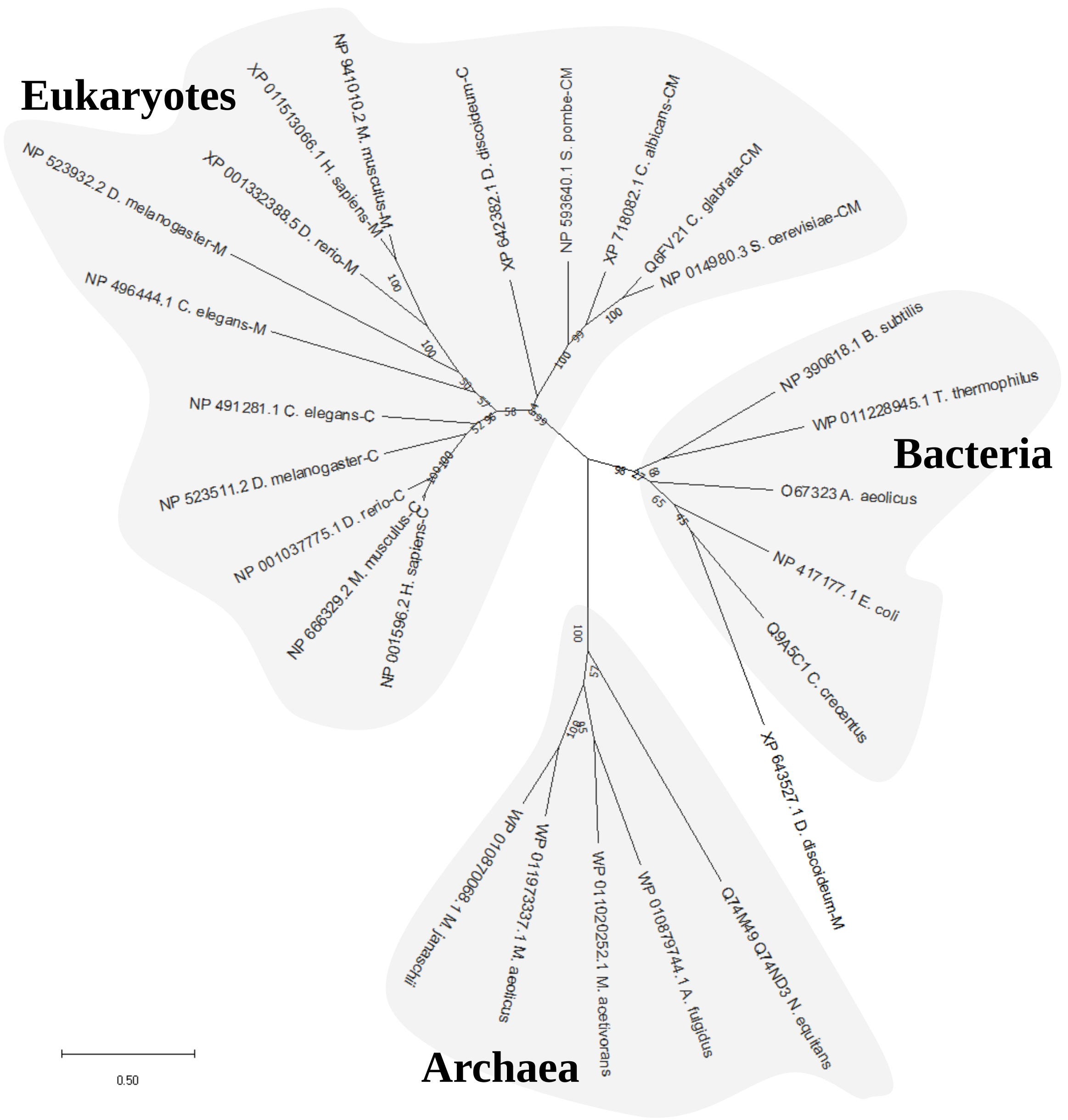

Figure S3. Evolutionary analysis of AlaRS by Maximum Likelihood method. The evolutionary history was inferred by using the Maximum Likelihood method and the Tamura–Nei model. The tree with the highest log likelihood (-1519.13) is shown. The percentage of trees in which the associated taxa clustered together is shown next to the branches. Initial tree(s) for the heuristic search were obtained automatically by applying the Neighbor–Join and BioNJ algorithms to a matrix of pairwise distances estimated using the Tamura–Nei model, and then selecting the topology with the superior log likelihood value. The tree is drawn to scale, with branch lengths measured in the number of substitutions per site. This analysis involved 30 nucleotide sequences. There was a total of 92 positions in the final dataset. Evolutionary analyses were conducted in MEGA X.

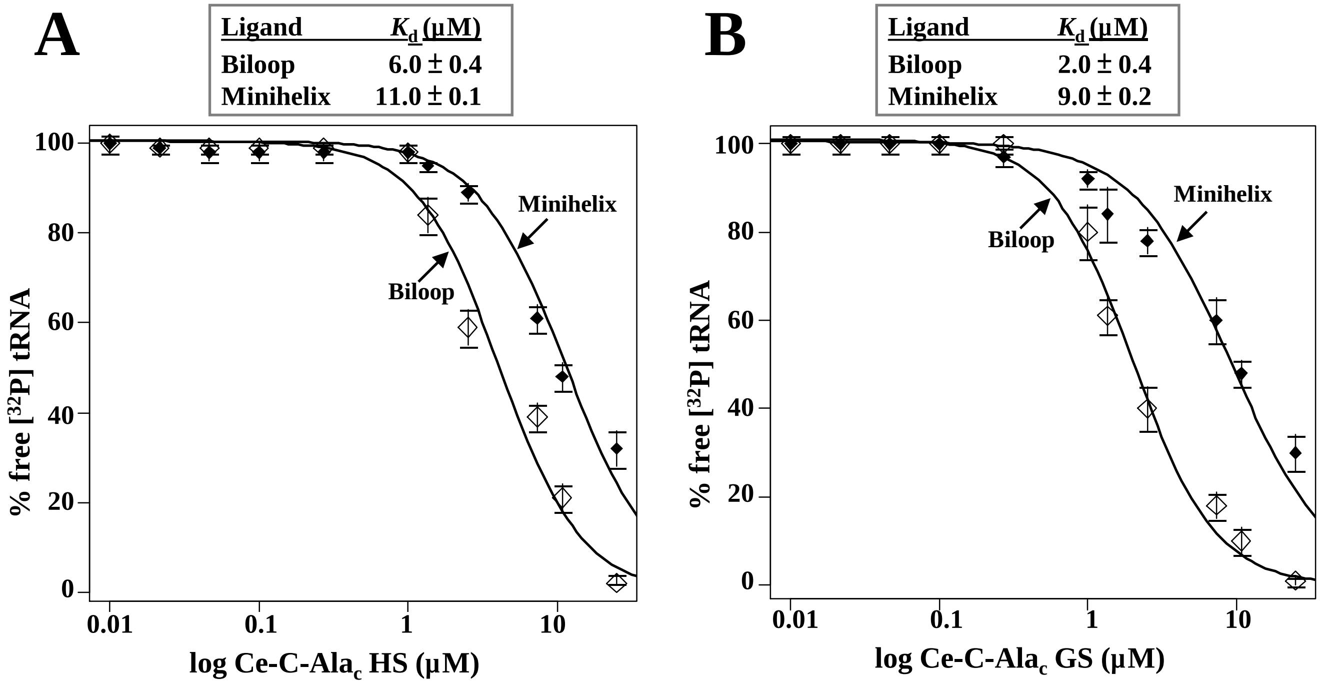

Figure S4. Mapping the tRNA-binding site of the Ce-C-Ala$_c$ subdomains. The binding affinities of the (A) helical and (B) globular subdomains of Ce-C-Ala$_c$ towards the minihelix and biloop of CetRNA$_n^{Ala}$ were determined by EMSAs with protein concentrations ranging from 32 to 0.125 µM.

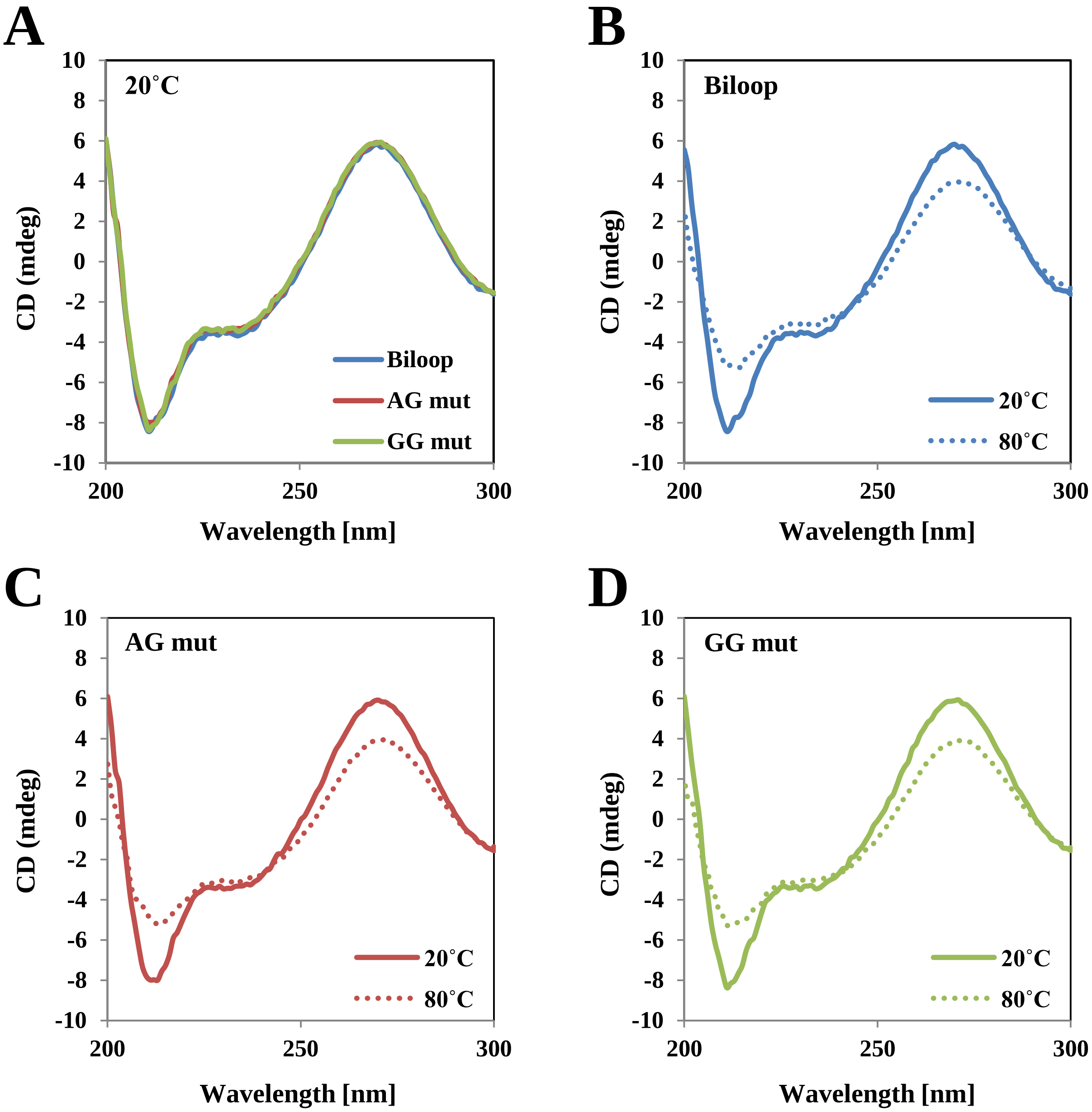

Figure S5. CD spectra of biloop. Wavelength scanning for (A) WT and mutant biloops at 20˚C, (B) WT biloop at 20˚C and 80˚C, (C) AG mut at 20˚C and 80˚C, and (D) GG mut at 20˚C and 80˚C.

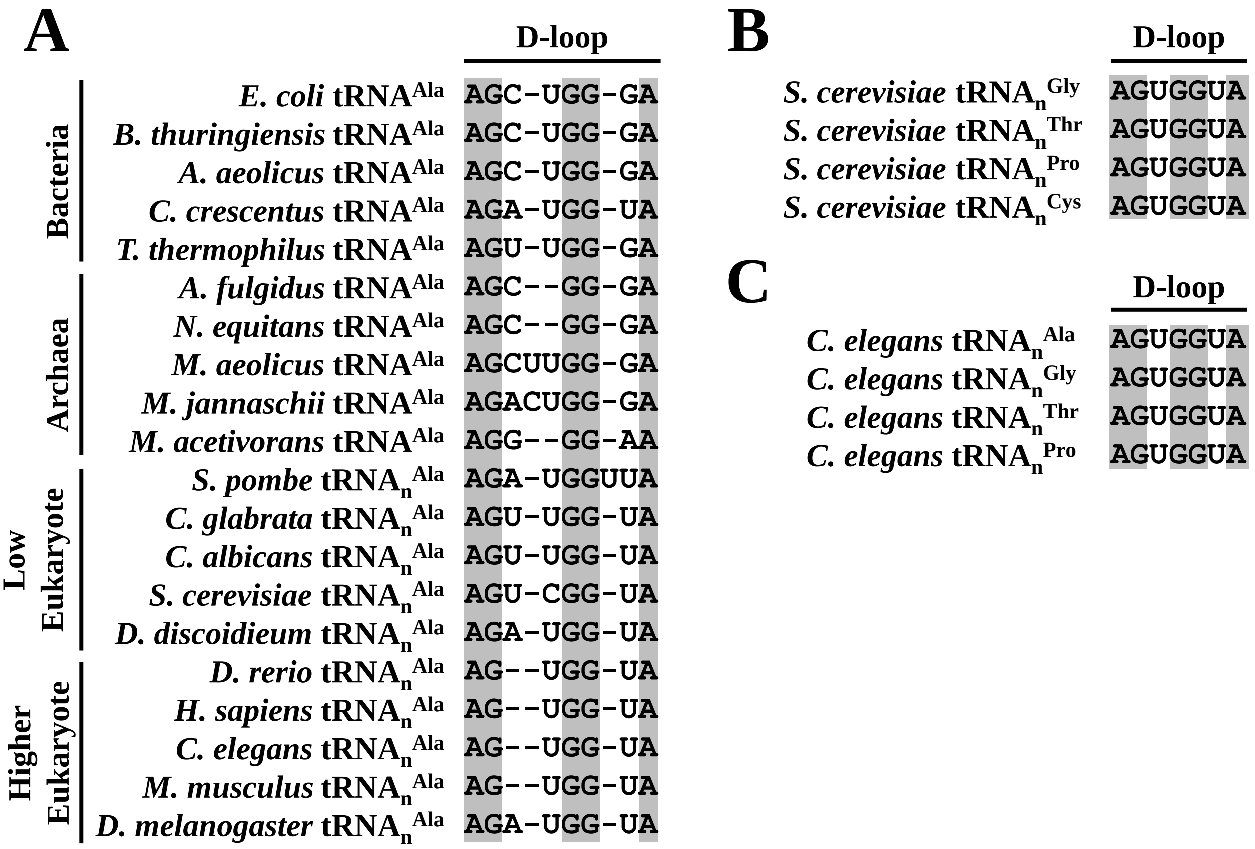

Figure S6. Alignment of the D-loop sequences. (A) Alignment of the D-loops of tRNAs^{Ala} retrieved from bacteria, archaea, and eukaryotes. (B) Yeast and (C) nematode tRNAs that possess the same D-loop sequence as CetRNA$_n^{Ala}$. The conserved invariant bases in the D-loop are shaded.

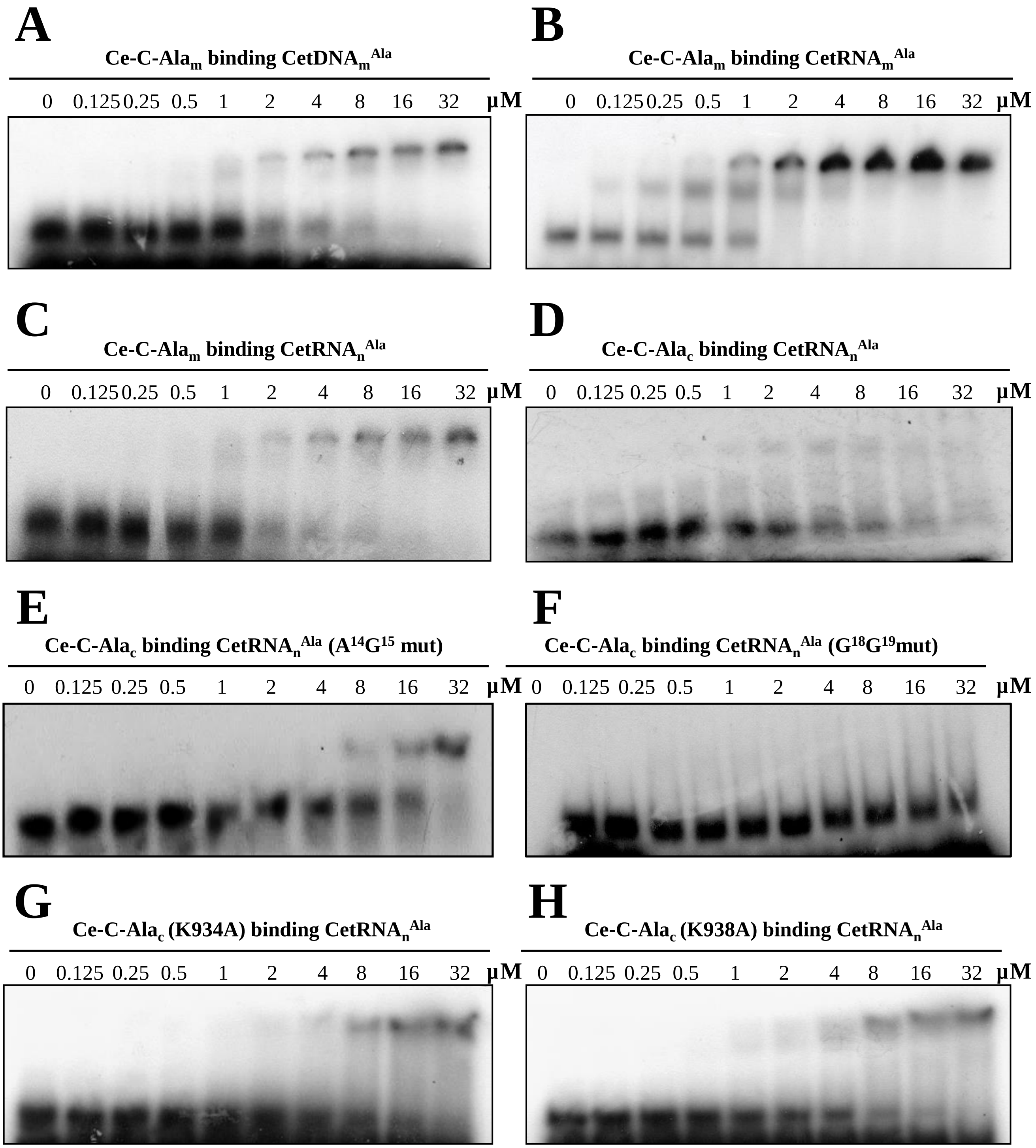

Figure S7. Representative EMSA figures. tRNA or DNA binding by C-Ala was determined by an EMSA. Here shows some of the representative EMSA figures.
